# Geographic disparities and temporal changes in risk of death from myocardial infarction in Florida, 2000–2014

**DOI:** 10.1186/s12889-019-6850-x

**Published:** 2019-05-03

**Authors:** Evah W. Odoi, Nicholas Nagle, Shamarial Roberson, Kristina W. Kintziger

**Affiliations:** 1 Comparative and Experimental Medicine, College of Veterinary Medicine, The University of Tennessee, 2407 River Drive, Knoxville, TN 37996 USA; 2 Department of Geography, The University of Tennessee, 2407 River Drive, Knoxville, TN 37996 USA; 3 Florida Department of Health, Bureau of Chronic Disease Prevention, 4052 Bald Cypress Way, Tallahassee, FL USA; 4 Department of Public Health, The University of Tennessee, 1914 Andy Holt Avenue, Knoxville, TN 37996 USA

**Keywords:** Myocardial infarction mortality, Geographic clusters, Disparities, Temporal trend

## Abstract

**Background:**

Identifying disparities in myocardial infarction (MI) burden and assessing its temporal changes are critical for guiding resource allocation and policies geared towards reducing/eliminating health disparities. Our objectives were to: (a) investigate the spatial distribution and clusters of MI mortality risk in Florida; and (b) assess temporal changes in geographic disparities in MI mortality risks in Florida from 2000 to 2014.

**Methods:**

This is a retrospective ecologic study with county as the spatial unit of analysis. We obtained data for MI deaths occurring among Florida residents between 2000 and 2014 from the Florida Department of Health, and calculated county-level age-adjusted MI mortality risks and Spatial Empirical Bayesian smoothed MI mortality risks. We used Kulldorff’s circular spatial scan statistics and Tango’s flexible spatial scan statistics to identify spatial clusters.

**Results:**

There was an overall decline of 48% in MI mortality risks between 2000 and 2014. However, we found substantial, persistent disparities in MI mortality risks, with high-risk clusters occurring primarily in rural northern counties and low-risk clusters occurring exclusively in urban southern counties. MI mortality risks declined in both low- and high-risk clusters, but the latter showed more dramatic decreases during the first nine years of the study period. Consequently, the risk difference between the high- and low-risk clusters was smaller at the end than at the beginning of the study period. However, the rates of decline levelled off during the last six years of the study, and there are signs that the risks may be on an upward trend in parts of North Florida. Moreover, MI mortality risks for high-risk clusters at the end of the study period were on par with or above those for low-risk clusters at the beginning of the study period. Thus, high-risk clusters lagged behind low-risk clusters by at least 1.5 decades.

**Conclusion:**

Myocardial infarction mortality risks have decreased substantially during the last 15 years, but persistent disparities in MI mortality burden still exist across Florida. Efforts to reduce these disparities will need to target prevention programs to counties in the high-risk clusters.

## Background

The rates of deaths from cardiovascular diseases (CVD), such as coronary heart disease (CHD) and myocardial infarction (MI), have decreased in the US in the last five decades [1]. However, CVD remain the leading cause of preventable premature deaths in the US, accounting for one in every four fatalities in the country [2]. MI, or heart attack, contributes significantly to this burden, with approximately 14% of the 790,000 people who experience an MI in the US each year dying from it [3].

Cardiovascular diseases also represent a serious economic burden to the US healthcare system, constituting 17% of national health expenditures in 2014 [3], with MI being the most expensive condition to treat [4]. The burden of MI is particularly high in the southeastern US states, including Florida, where 5.3 and 12% of the adult and elderly (≥65 years) populations, respectively, reported a history of acute MI in 2014 [5]. Moreover, the increase in mean age of the population coupled with an upsurge in risks of obesity and type 2 diabetes [2] are expected to exacerbate the burden of MI and increase its public health and economic costs [6].

Consistent with the trends seen nationally [1], an overall decline in MI/ischemic heart disease mortality risks has been observed in Florida [7, 8]. However, it has been shown that population subgroups defined by geography and other factors may show widening disparities in cardiovascular health, despite reductions in overall CVD mortality risks [9]. Additionally, previous studies showing geographic disparities of MI mortality risks at county- [10] and census tract-levels [11, 12] suggest that geographic hotspots of MI mortality risks may exist in Florida. Therefore, it is strategically advantageous to identify populations with high MI burdens and investigate how the MI burdens change over time to guide control programs geared towards reducing/eliminating disparities and improving population health. Moreover, understanding how MI burdens change over time may reveal the effectiveness of intervention programs and can be used to guide policy decisions and resource allocation. Unfortunately, no rigorous population-level studies have been conducted to determine if the decreases in MI mortality risks have occurred equitably across all communities in the state. Therefore, our objectives were to: (a) investigate the spatial distribution and clusters of MI mortality risk in Florida; and (b) assess temporal changes in geographic disparities in MI mortality risks in Florida from 2000 to 2014.

## Methods

### Study design and study population

This is a retrospective ecological study using Florida MI mortality data for the period 1/1/2000–12/31/2014. The study population included all deceased Florida residents whose underlying cause of death was listed as MI, according to the *International Classification of Diseases*, tenth revision: ICD-10 Code(s): I21 (acute myocardial infarction) and I22 (subsequent myocardial infarction). The variables of interest included age, county of residence, and year of death. We used the county as the geographic unit of analysis.

### Data sources and data preparation

We obtained county-level MI mortality data for the age-groups 0–34, 35–44, 45–54, 55–64 and ≥ 65 year-olds covering the 2000–2014 time period from the Florida Department of Health (DOH) website [7]. Due to a small number of deaths (< 25 events) in some counties, DOH routinely pools age-specific MI death counts by three-year intervals to help stabilize death risks and to maintain patient anonymity and confidentiality.

We also obtained county-level annual population estimates for age categories matching the MI mortality data (i.e., 0–34, 35–44, 45–54, 55–64 and ≥ 65 year-olds) from DOH [13] and used this as denominator data for calculating age-specific mortality risks. We downloaded county-level cartographic boundary shape files for all cartographic displays from the US Census Bureau website [14].

### Descriptive statistics

MI mortality risks per 100,000 population were calculated and directly age-standardized to the 2000 US Standard Population [15] in SAS v.9.4 (SAS Institute; Cary, NC). Despite pooling death counts by three-year intervals to address the small number problem, a number of rural counties still had < 25 MI-deaths. According to Curtin and Klien [16], such areas are considered small areas; hence, unsmoothed age-adjusted risks from these areas would be highly unstable due to high variances. Therefore, to minimize the impact of the high variances and adjust for spatial autocorrelation (i.e. clustering), we computed Spatial Empirical Bayes (SEB) smoothed risks using 1st order queen weights in GeoDa [17]. All descriptive analyses were done in SAS v.9.4 (SAS Institute; Cary, NC).

### Investigation of spatial clusters

We investigated circular spatial clusters of high MI mortality risks using Kulldorff’s circular spatial scan statistics (CSSS) implemented in FlexSCcan v 3.1.2, using age-adjusted MI mortality counts and a Poisson probability model specifying restricted likelihood ratio test (RLRT) to preclude absorption of counties with non-elevated risks into high-risk clusters [18]. We specified an alpha of 0.2 [19] and a maximum spatial cluster size of 34 counties, which corresponds to about half the number of counties in Florida. Additionally, we identified non-circular spatial clusters using Tango’s flexible spatial scan statistics (FSSS) specifying a Poisson probability model again with a RLRT [20], an alpha of 0.2 and 34 counties as the maximum spatial cluster size. The FSSS generates irregularly shaped windows and is well suited for irregularly shaped areas such as along Florida’s rivers, lakes, and coastline. Clusters occurring in such areas would not be detected by the CSSS. We computed the mortality risks in significant (*p* < 0.05) clusters as the product of standardized mortality ratios and the crude MI mortality risk for Florida.

We investigated circular spatial clusters of low MI mortality risks using CSSS, implemented in SaTScan v8.0 software. We used a discrete Poisson probability model while adjusting for age as a confounder and specifying non-overlapping, circular, purely spatial clusters of low risks. A maximum window size of 13.4% of Florida’s population was used. This choice was based on the population of the largest county (Miami-Dade) to ensure that every county had a chance of being a cluster, while also minimizing the chance of identifying unrealistically large clusters that could comprise counties with high and/or non-elevated risks. Statistical inference was based on likelihood ratio test (LRT), and the *p*-value was obtained through 999 Monte Carlo replications. Statistical significance was assessed at an alpha of 0.05.

### Cartographic display

We used ArcGIS Version 10.3.1 (ESRI, 2010) to perform all GIS manipulations, and to display all significant biologically meaningful clusters. Jenk’s optimization classification scheme was used to determine the intervals for displaying SEB risks as choropleth maps. According to Prates et al. [21], spatial scan statistics has low power to detect clusters in low population density areas. Consequently, the relative risks (RR) for the spatial scan statistic may have an upward (for high risk clusters) or downward (for low risk clusters) bias, particularly when the population at risk is small. Accordingly, sparsely populated rural areas require a high RR to accurately detect the correct high-risk cluster, and a low RR to correctly detect low-risk cluster. Therefore, we considered significant high-risk clusters identified in rural and urban counties to be meaningful if the RR value was ≥1.3 and ≥ 1.2, respectively. On the other hand, we considered significant low-risk clusters identified in rural and urban counties to be meaningful if the RR value was ≤0.7 and ≤ 0.8, respectively.

### Temporal changes

We plotted mortality risks against time to examine the temporal trends, and calculated percentage change in mortality risks during the study period by computing the difference between the 2000 and 2014 risks and dividing the result by the 2000 risk. We assessed spatial disparities in MI mortality risks by comparing the magnitude of excess risks in high-risk clusters at the beginning and at the end of the study, using the low-risk cluster with the lowest MI mortality risks as the baseline.

## Results

There were 58,198 MI deaths in Florida between 2000 and 2014. The overall annual age-adjusted MI mortality risks were 55.5 (2000–2002), 43.8 (2003–2005), 33.1 (2006–2008), 29.8 (2009–2011), and 28.1 (2012–2014) deaths/100,000 population over the study period. This represented an overall decrease of 48% in MI mortality risks during the period of interest.

### Spatial patterns

**Fig. 1:**
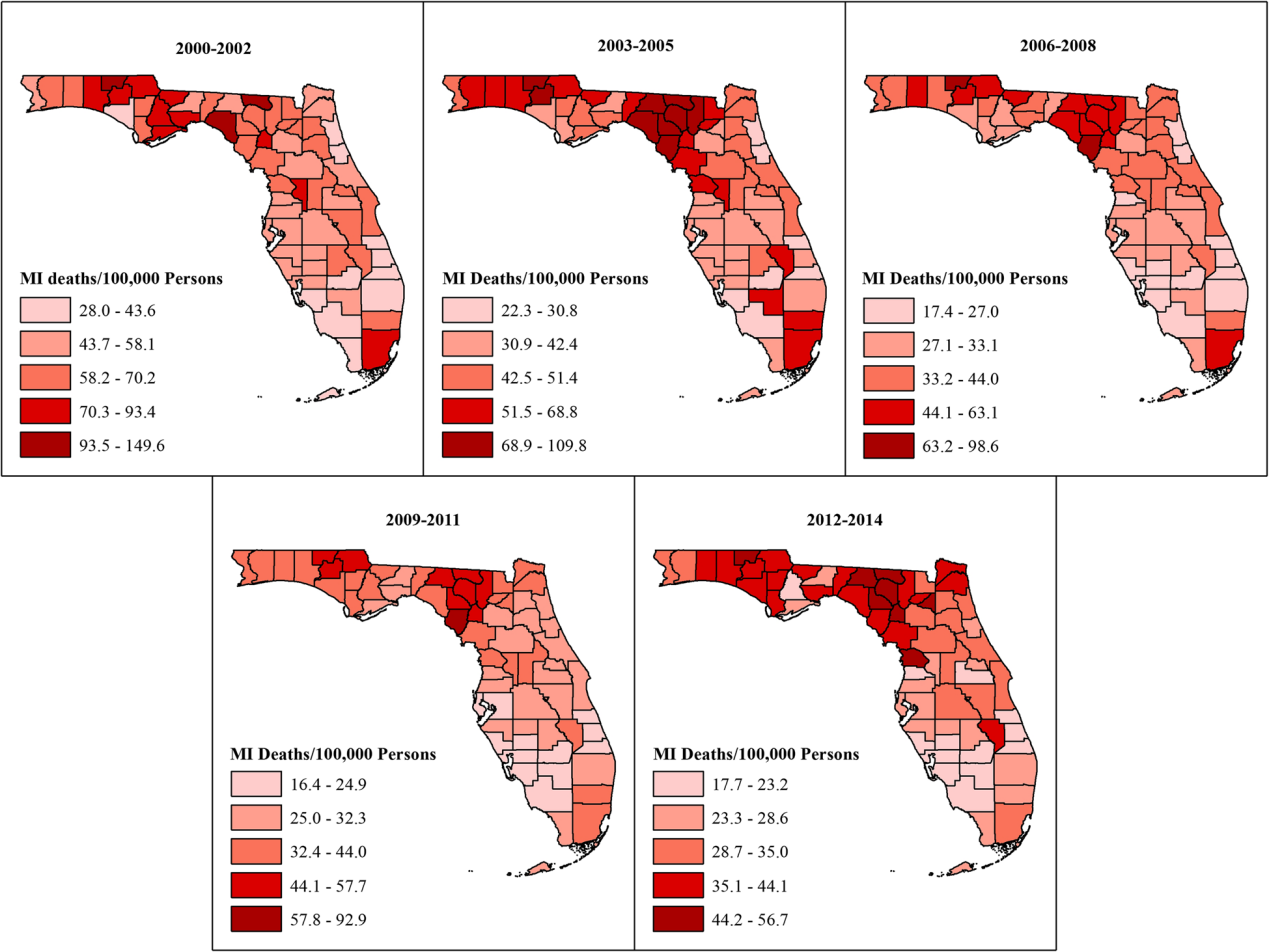
County-level age-adjusted Spatial Empirical Bayes smoothed myocardial infarction risks in Florida, 2000–2014

The temporal changes in geographic distribution of SEB risks are shown in Fig. 1. The risks declined during the study period and ranged from 28.1–149.6 deaths/100,000 population at the beginning of the study to 17.7–56.7 deaths/100,000 population at the end of the study. Although the risks decreased throughout the state during the study period, counties in the north had consistently higher MI mortality risks than those in the south. There was also a clear urban-rural divide, with the rural north having the highest risks and the urban south having the lowest risks throughout the study period. Moreover, the proportion of northern counties in the two highest quintiles increased from 16% in 2000–2002 to 36% in 2012–2014. No such changes were visible in the south.

#### Kulldorff’s circular spatial clusters (CSSS)

**Fig. 2:**
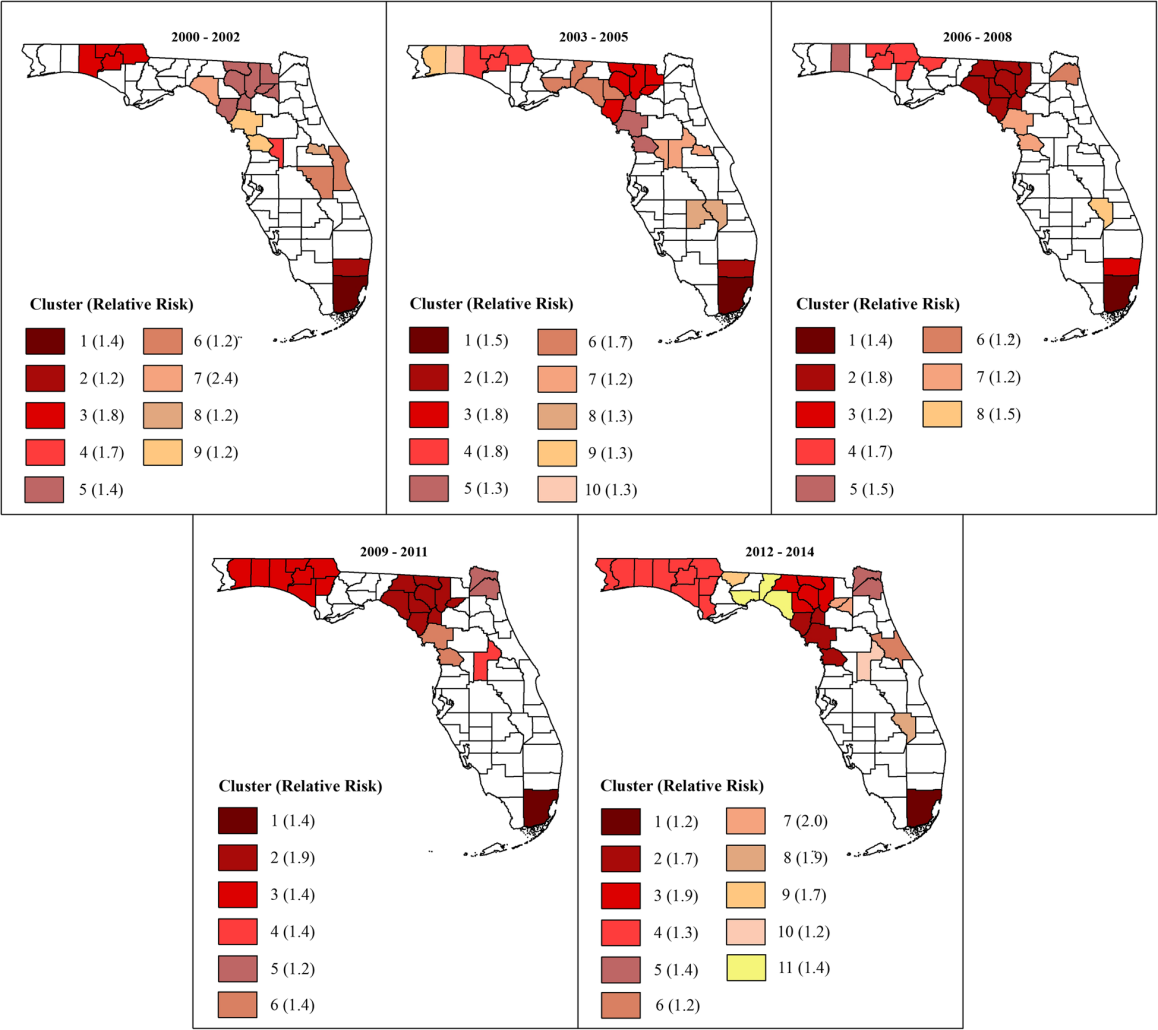
Spatial circular clusters of high myocardial infarction mortality risks in Florida, 2000–2014

**Fig. 3:**
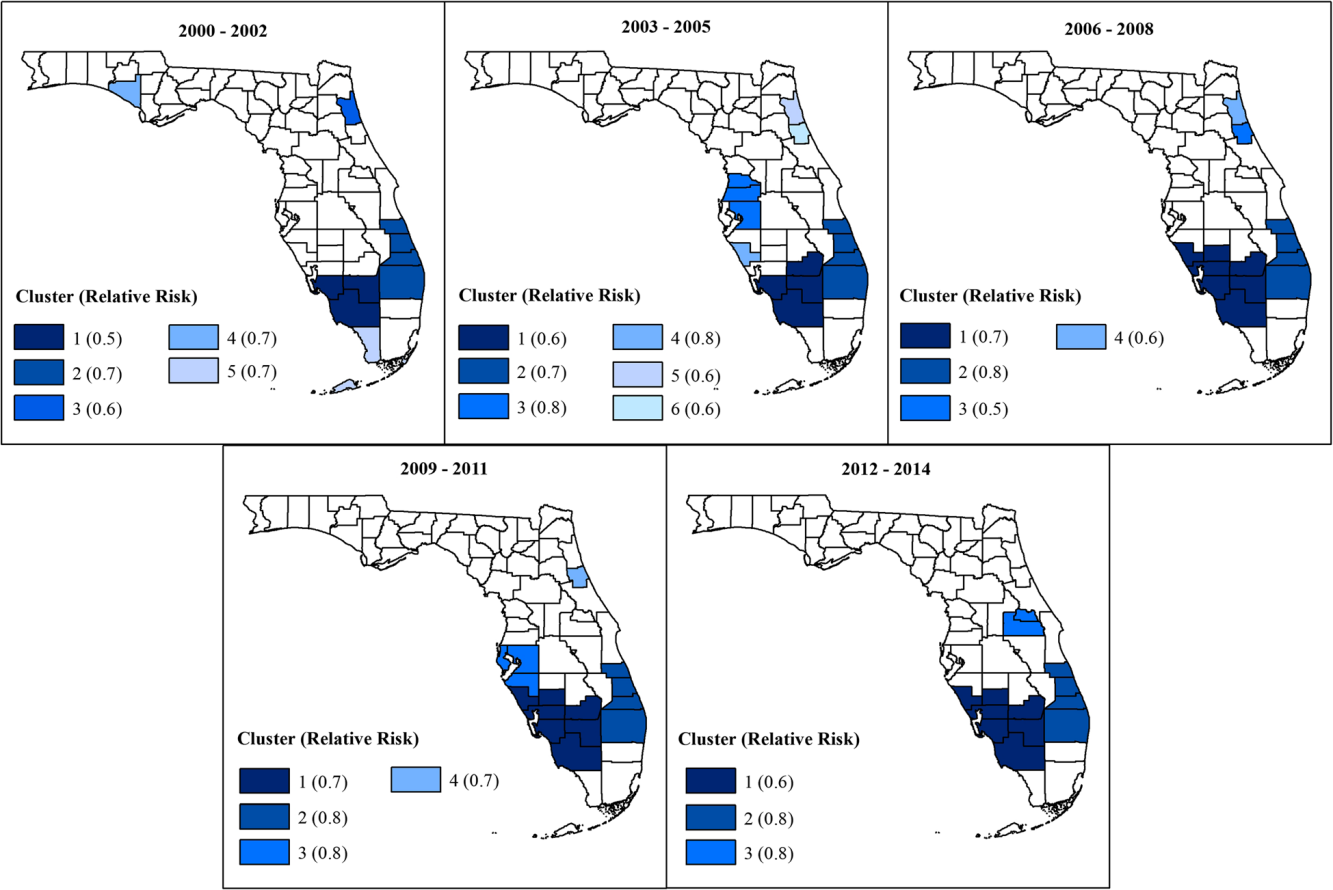
Spatial circular clusters of low myocardial infarction mortality risks in Florida, 2000–2014

**Fig. 4:**
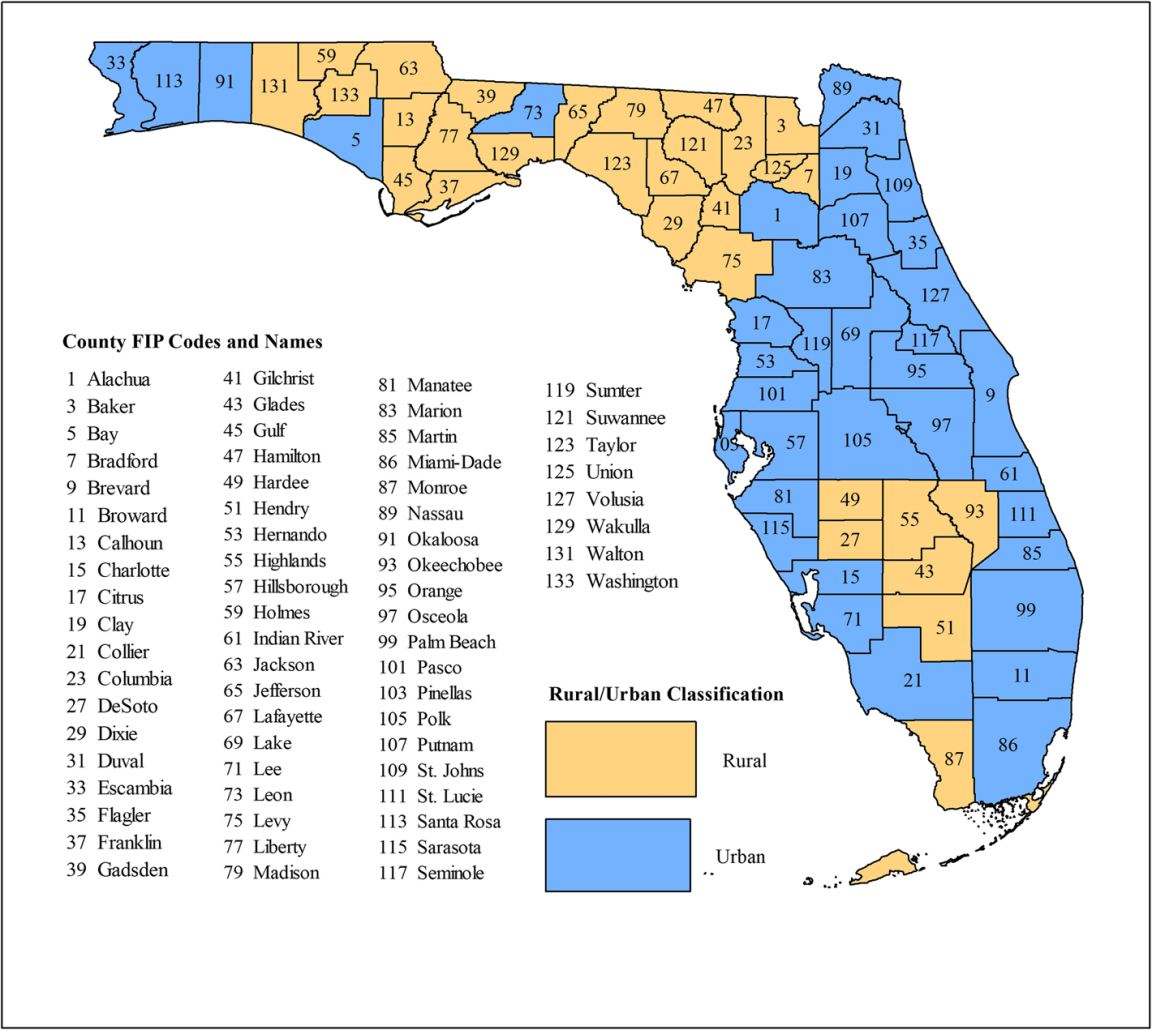
Florida counties and their rural/urban classification

Figures 2 and 3 show the geographic distribution circular spatial clusters of high and low MI mortality risks. Consistent with the visual patterns of SEB smoothed risks (Fig. 1), the Kulldorff’s CSSS identified large clusters of high MI mortality risks predominantly in the North (Fig. 2) and large low-risk clusters predominantly in South Florida (Fig. 3). A total of 6–11 high-risk clusters were identified during each of the three-year time intervals between 2000 and 2014. The largest high-risk clusters were located in northwest and north central parts of Florida (Fig. 2), which are predominantly rural (Fig. 4) based on the Florida Department of Health Office of Rural Health definition of rural areas i.e. population density < 100 people/sq. mile [22]. Smaller high-risk clusters were identified in Central, West Central, Northeast, and Southeast Florida, with the urban high-risk cluster in Miami-Dade County being the most prominent (Fig. 2). A total of 3–6 low-risk clusters, were identified. Large low-risk clusters were located mostly in urban counties in the southeast and southwest (Figs. 2 and 3). A few smaller clusters were identified in Northwest, Northeast, Central, and West Central Florida.

Figures 2 and 3 also show that 4–5 high-risk clusters and 2 low-risk clusters persisted throughout the study period. Clusters with persistently high mortality risks were located in the Northwest, North Central and Southeast Florida. Counties that persisted in the high-risk clusters in the northwest included Holmes, Jackson, Washington counties. Walton County was part of that cluster in all the three-year time intervals with the exception of the 2006–2008 period. Two persistent high-risk clusters were identified in North Central Florida. The larger cluster comprised Columbia, Dixie, Gilchrist, Hamilton, and Suwannee counties, and the smaller cluster comprised Citrus and Levy counties. The Miami-Dade cluster also persisted throughout the study period. Counties that persisted in the low-risk cluster in Southeast Florida included Indian River, St. Lucie, Martin, and Palm Beach. Collier, Hendry and Lee counties persisted in the low-risk cluster in Southwest Florida.

Substantial changes in cluster status occurred in North and Central Florida, with several counties that were not a part of any cluster at the beginning of the study transitioning to high-risk clusters by the end of the study. These included Calhoun, Duval, Escambia, Gulf, Lafayette, Madison, Nassau, Okaloosa and Wakulla counties in North Florida and Lake, Okeechobee, and Volusia counties in Central Florida. The opposite trend was also observed, where some counties in Central (Brevard, Osceola, and Sumter) and Southeast Florida (Broward) transitioned from high-risk clusters at the beginning to not being part of any cluster at the end of the study. Transitions of counties to low-risk clusters were less frequent, with only Seminole County in Central Florida transitioning from a high- to low-risk cluster, and Charlotte, DeSoto, Glades, and Sarasota counties in Southwest Florida transitioning from no-cluster to low-risk cluster. The lone low-risk cluster identified in Northwest Florida in Bay County in the 2000–2002 period transitioned to a high-risk cluster by the 2012–2014 period. There were considerable variations in relative risks (RR) among the clusters, ranging from 1.2 to 2.4 among the high-risk clusters, and from 0.5 to 0.8 among low-risk clusters.

#### Tango’s circular and non-circular spatial clusters (FSSS)

**Fig. 5:**
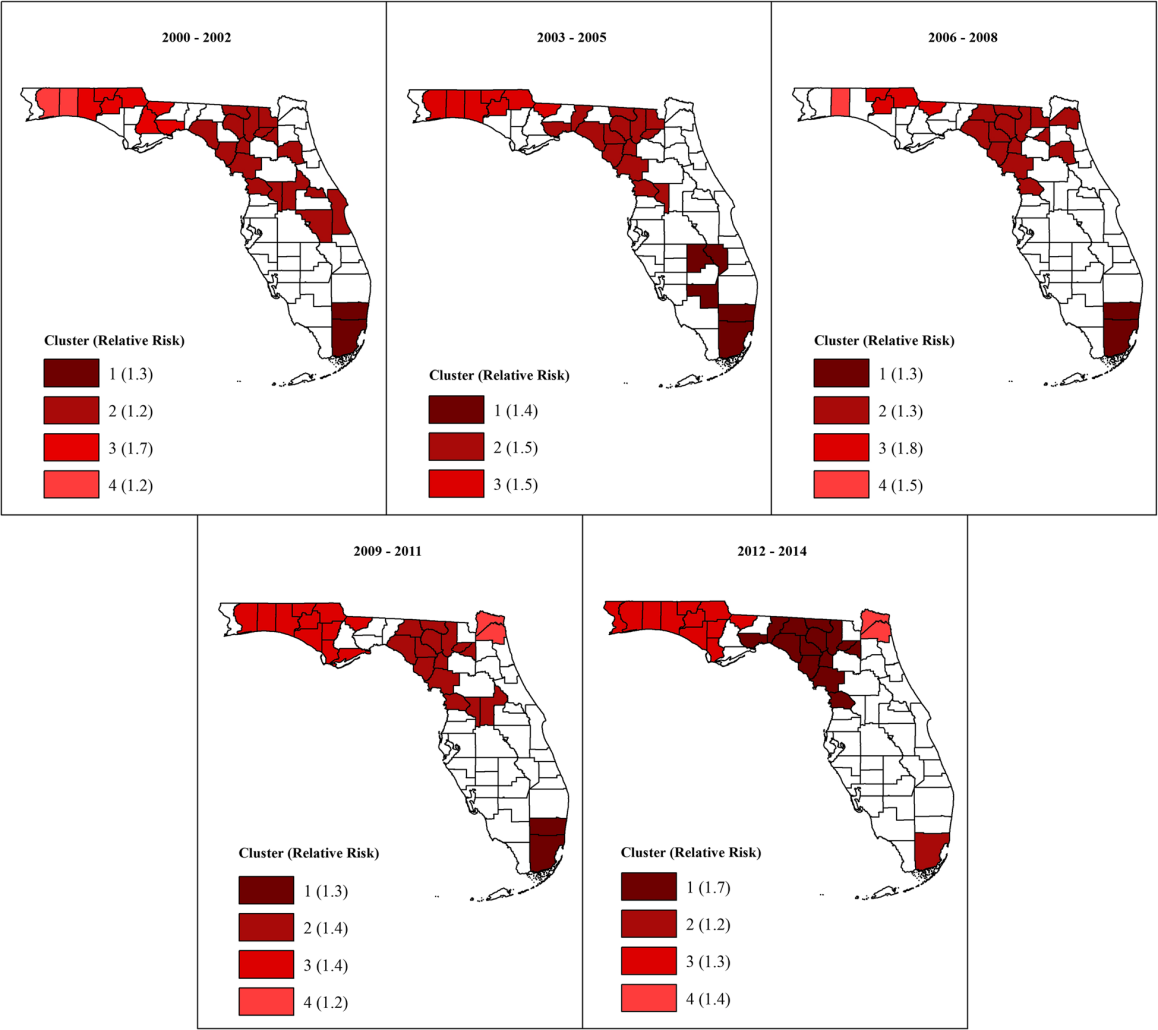
Circular and non-circular spatial clusters of high myocardial infarction mortality risks in Florida, 2000–2014

The geographic distributions of high-risk circular and non-circular clusters identified using Tango’s flexible spatial scan statistics are presented in Fig. 5. While the location of clusters and the general patterns of clustering of MI risks identified using Tango’s FSSS (Fig. 5) mirrored those of clusters identified using Kulldorff’s CSSS (Fig. 2), fewer clusters were identified using FSSS (3–5 clusters) than CSSS (6–11 clusters). The FSSS also resulted in larger clusters, often comprising all counties identified using CSSS plus additional counties. The RR among clusters identified using FSSS were lower than those identified using CSSS (Fig. 5).

### Temporal changes

**Fig. 6:**
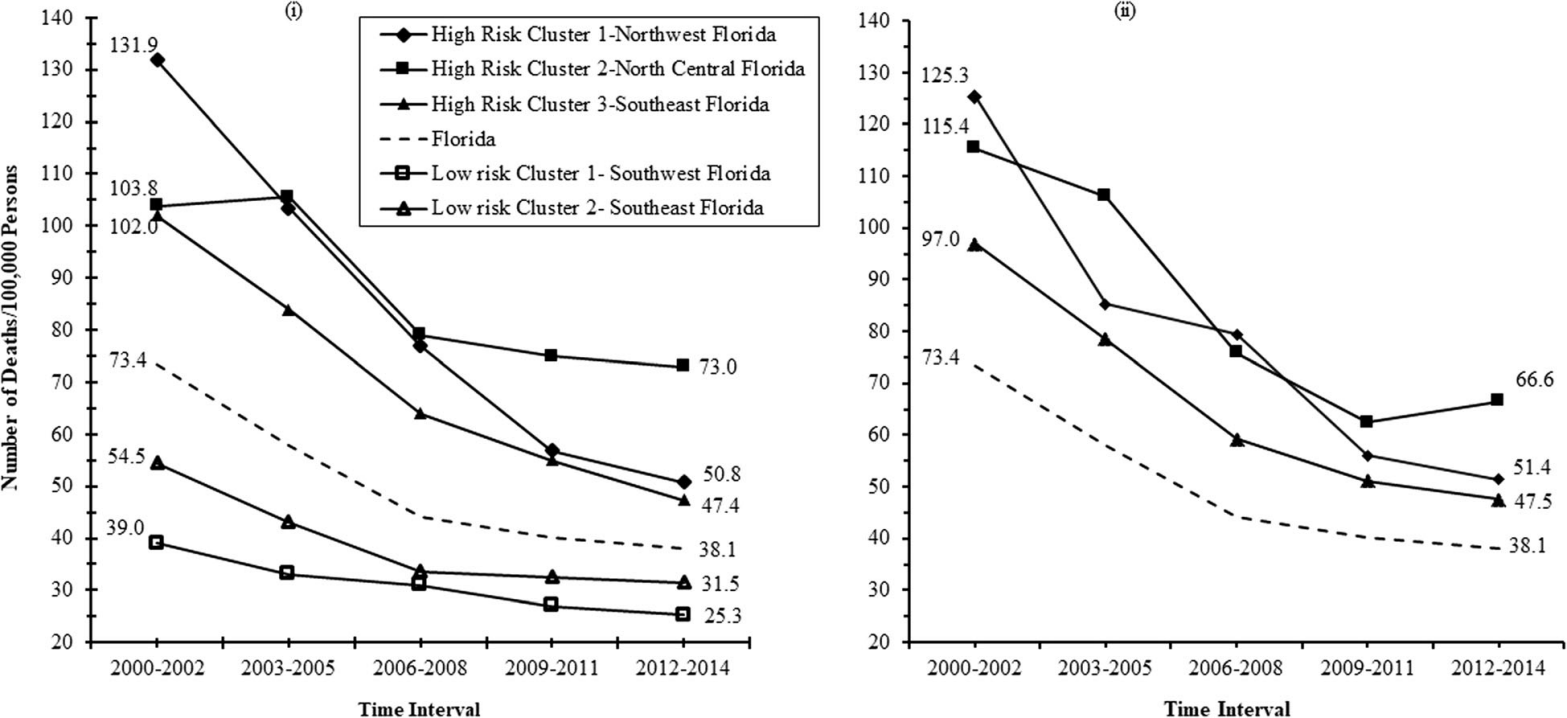
Changes in annual myocardial infarction mortality risks in persistent high- and low-risk (**i**) Kulldorff’s circular and (**ii**) Tango’s circular and non-circular spatial clusters, Florida 2000–2014

The temporal changes in MI mortality risks among persistent CSSS clusters are shown in Fig. 6. Overall, MI mortality risks decreased by 48% which is equivalent to an average rate of decline of 3.2%/year. MI mortality risks decreased more rapidly (4.1%/year) between 2000 and 2008, after which (2009–2014) they decreased by a meagre 0.8%/year.

Declines in MI mortality risks showed considerable variation among clusters and ranged from 35 to 42% in low-risk clusters and from 30 to 61% in high-risk clusters. This resulted in average rates of decline of 2.3–2.8%/year and 2.0–4.1% per year in low- and high-risk clusters, respectively. It is interesting to note that mortality risks in the high-risk cluster in North Central Florida decreased at a lower rate (2.0%/year) than in the two low-risk clusters (2.3–2.8%). Similar to the temporal pattern observed for the entire state, there were more dramatic declines in mortality risks in both high- (2.7–4.6%/year) and low-risk (2.3–4.3%/year) clusters during the first nine years of the study. Thereafter, the rates of decline slowed to 0.4–2.3%/year, with the high-risk cluster in North Central Florida showing the slowest rate of decline despite having the highest MI mortality risk.

The patterns of temporal changes in MI mortality risks in high-risk circular and non-circular FSSS clusters that persisted during the study period (Fig. 6) are generally similar to the patterns observed for high-risk CSSS circular clusters. The largest decline occurred in the high-risk cluster in Northwest Florida (59%), followed by the high-risk cluster in Southeast Florida (51%) and then the high-risk cluster in North Central Florida (42%). As with CSSS clusters, MI mortality risks decreased rapidly during the first nine years of the study, after which they declined at a substantially lower rate. There are early signs that MI mortality risks in the high-risk cluster in North Central Florida could be on an upward trend.

Generally, MI mortality risks decreased more rapidly in high- than in low-risk clusters during the early portion of the study (2000–2008), and at a similar rate in both high- and low-risk clusters thereafter (2009–2014). This resulted in lower disparities in MI mortality risks between high- and low-risk clusters at the end than at the beginning of the study period (Fig. 6). For instance, the risk difference (RD) between the high-risk cluster in Northwest Florida and the referent low-risk cluster in the Southwest Florida decreased by 72.5% from 92.9 deaths/100,000 persons in 2000–2002 to 25.5 deaths/100,000 persons in 2012–2014. The RD between the high-risk cluster in Southeast Florida and the referent low-risk cluster showed a relatively similar reduction, decreasing by 65% from 63 deaths/100,000 persons at the beginning of the study to 22.1 deaths/100,000 persons at the end of the study. The RD between the high-risk cluster in North Central Florida and the low-risk cluster in Southwest Florida decreased by 26% from 64.8 deaths/100,000 persons at the beginning of the study period to 47.7 deaths/100,000 persons at the end the study.

In spite of the impressive declines, annual MI mortality risks for the high-risk clusters in Northwest and Southeast Florida at the end of the study period (47.4–50.8 deaths/100,000 persons) were at par with mortality risks observed in the low-risk clusters at the beginning of the study period (39–54.5 deaths/100,000 persons). This implies that MI mortality risks for counties in high-risk clusters lagged behind those for counties in low-risk clusters by 1.5 decades. Moreover, the annual MI mortality risk observed in the high-risk cluster in North Central Florida at the end of the study period (73 deaths/100,000 persons) was substantially higher than the risk for the referent low-risk clusters (39 deaths/100,000 persons) at the beginning of the study period. Thus, counties in the high-risk cluster in North Central Florida lagged behind counties in the low-risk clusters by over 1.5 decades.

## Discussion

We investigated geographic distribution and spatial clusters of MI mortality risks in Florida over a period of 15 years. We also identified communities with consistently high MI burden over the study period. Study findings will be useful for guiding resource allocation for intervention programs. Florida has a racially and ethnically diverse population with large proportions of minority, immigrant, and elderly populations, hence it foreshadows the demographic structure projected for the US population by the year 2030 [23]. Therefore, Florida’s strategy to address the high MI burden will not only be critical to Florida’s future, but it will be instructive for the rest of the US.

Similar to other studies using county-level data to assess cardiovascular mortality disparities across the US [8, 10], this study found disparities in the burden of MI across Florida, with the north having the highest mortality risks while the south had the lowest risks. This is consistent with the shift in the concentration of counties with high rates of heart disease-related mortality from Northeastern US to socioeconomically disadvantaged areas in the Deep South that was observed by Casper et al. [10] over a 40-year period.

The identification of high-risk clusters mainly in rural north and low-risk clusters almost exclusively in urban south suggests that different segments of Florida’s population have not benefitted equitably from preventive and treatment efforts. Moreover, these findings mirror those of stroke mortality risks in Florida between 1992 and 2012 [24]. Other studies have also reported disparities in MI/heart disease-related mortality risks in southeastern United States based on rurality. For instance, Casper et al. [10] also identified a large persistent low-rate cluster of heart disease mortality in urban counties in southern Florida and 1–2 high-rate clusters in the rural north between 1972 and 2010. Roth et al. [8], also reported clustering of low risks of CVD and ischemic heart disease mortality in South Florida counties and clustering of high risks in North Florida counties in 2014. Odoi and Busigye [12] reported higher MI-mortality risks in rural than in urban neighborhoods in middle Tennessee. Higher mortality rates for CHD, the principal cause for MI, have also been reported for rural/non-metro areas compared to urban/metro areas in southern US [25]. By contrast, Pedigo et al. [11] reported higher odds of urban and suburban neighborhoods being in a high-risk cluster than rural neighborhoods.

We did not investigate the determinants of the identified geographic disparities. However, based on findings from previous studies, the disparities may be associated with disparities in distribution of MI risk factors and access to preventive and treatment services. For instance, rural communities generally have lower prevalence of physical activity [26] and good dietary habits [27] compared to urban populations. Moreover, increased mechanization and automation of farm work has reduced the amount of physically demanding occupations in rural areas [28], making rural lifestyle more sedentary [29]. These contribute to higher risks of obesity, hypertension and diabetes which lead to higher MI-mortality risks in rural than urban areas. By contrast, the prevalence of nonsmoking, normal body weight, and physical activity, etc., are higher in urban than rural counties in US [30].

Most North Florida counties are rural, sparsely populated, medically underserved [31, 32] and have low rates of health insurance coverage [33]. Since health funding is allocated based on population, rural counties tend to have limited resources for adequate prevention and management of CVD and its risk factors [34]. The distribution of health workforce is also geographically skewed, with rural counties having inadequate supply of general practitioners [35] and cardiac specialist [36]. Moreover, cardiac centers tend to be clustered in urban center [37], leading to long travel times and poor MI outcomes.

Socioeconomic status (SES) is one of the most reliable predictors of cardiovascular health disparities, with people of low SES experiencing higher mortality from MI and other cardiovascular health outcome [38]. Clustering of CVD risk factors has been reported among US residents with low SES [39]. Socioeconomic status may also contribute to disparities in MI mortality risks by shaping exposure to unhealthy behaviors during childhood [40]. Since a majority of counties in North Florida have poor socioeconomic conditions [41], it is likely that lower SES for rural residents made them less likely to adopt and, therefore, benefit from improvements in prevention and control programs for MI [42], contributing to higher MI mortality risks in rural areas.

The composition of the populations in the different geographic regions is an important factor that may have also contributed to the disparities in MI mortality risks. North Florida has a higher proportion of African-Americans than the rest of Florida [43]. African-Americans tend to have higher burdens of MI [44] because they are less likely to receive certain cardiovascular interventions than whites [45] and as a result of stressors associated with systematic segregation in socioeconomically deprived neighborhoods during critical life stages [46]. In addition to traditional MI risk factors, environmental exposures such as higher, more variable temperatures in the north than the south [47], may have contributed to higher MI mortality risks in the north [48].

The identification of the lone high-risk cluster in Miami-Dade County was surprising because unlike other persistent high-risk clusters, it occurred in an urban county with a relatively younger population compared to Florida. Additionally, unlike the other persistent high-risk clusters, the Miami-Dade cluster was not identified in earlier county-level studies investigating geographic disparities in heart disease [10] and ischemic heart disease [8] in the US. However, the county has a high prevalence of other major risk factors for MI including hypertension (32.6%), high blood cholesterol (32.2%) overweight/obesity (87.2%), and physical inactivity (56.7%) [49]. Additionally, Miami-Dade County has a high proportion of socioeconomically-disadvantaged immigrant minority uninsured/underinsured population [50]. However, despite the high prevalence of MI risk factors and high under/uninsured rates, utilization rates for low-cost health care programs, such as the Federally Qualified Health Centers, are very low [50]. Therefore, low levels of utilization healthcare services and poorer control of hypertension and other modifiable risk factors for MI may also explain the presence of this cluster.

The reasons for the persistence of some counties in high- or low-risk clusters throughout the 15-year study period are not clear. However, persistence may be reflective of a lack of temporal changes in the geographic patterns for MI risk factors such as prevalence of cigarette smoking [51], hypertension [52], obesity, physical inactivity [53] and socioeconomic factors [54] reported in US counties.

The observed declines in MI mortality risks during the study period imply that population-wide preventive and control efforts to reduce the MI burden have had positive impacts across Florida [55]. These findings are consistent with those of other studies in the US that have shown steady declines in overall MI/CHD-related deaths at the national [56] and regional levels [57]. That a reduction in the prevalence of major risk factors contributed to reduced MI mortality risks in Florida was partly corroborated by a study that reported an 8.8% reduction in MI mortality rates in the state in 2004 following the implementation of the smoke-free ordinance in 2003. Three years prior to the ordinance, the rates declined at only 6.4% per year [58]. However, persistent clustering of MI-mortality risks, coupled with differences in rates of declines among clusters and over time indicate that geographic disparities still exist.

Disparities in geographic patterns and magnitude of rates of declines in MI mortality risks suggest that factors influencing the rates of MI mortality decline are not equitable across the state. According to Phelan et al. [42], the differential rates of decline in MI mortality risks among clusters may be related to disparities in access to social resources that influence adoption and/or the ability to benefit from improvements in MI prevention and control strategies.

The observed decline in MI mortality risks represents remarkable progress in reducing the burden of MI across Florida and is encouraging. However, in light of the fact that elimination of health disparities is one of the goals of the Healthy People 2020 national public health agenda [59], the levelling off of rates of declines from 2009 to 2014 is concerning. Thus, the goal of reducing CVD deaths by 20% by 2020 appears elusive. It is interesting to note that these results mirror the recent temporal trends reported for heart disease deaths in the US. For instance, Ma et al. [60] reported an annual rate of decline of heart disease deaths of 3.9% from 2000 to 2010, and a much slower annual rate of 1.4% from 2010 to 2013. Sidney et al [61]. reported annual rates of decline of CVD mortality of 3.79 and 0.65% between 2000 and 2011 and 2011–2014, respectively. Cardiovascular disease death rates decreased at an average of 3.7% per year between 2000 and 2011 and at less than 1%/year between 2012 and 2014, after which the rates actually increased by 1% in 2015 [4]. A deceleration in decrease in CHD mortality rates in the US was also reported between 2012 and 2015 [62]. These changes in the trajectory of MI and heart disease burden may be due to slowed progression in the favorable trends of MI prevention and/or treatment, coupled with an aging population and dramatic increases in the risks of obesity, hypertension, and diabetes mellitus over the past 25 years [2]. Capewell et al. [63] showed that improvements in survival among CHD patients in the US associated with decreases in the prevalence of CHD risk factors in the wider population were partially offset by increases in the prevalence of obesity and diabetes.

The fact that MI mortality risks for high-risk clusters at the end of the study (2012–2014) were at par with, or higher than the risks in low-risk clusters at the beginning of the study (2000–2002 period) indicates that counties in high-risk clusters lagged behind those in low-risk clusters in the south by at least 1.5 decades in reducing MI-mortality risks. Assuming a continuing downward trend, this implies that high-risk counties would require at least 15 additional years to achieve mortality risks seen in low-risk counties during the 2012–2014 period.

### Strengths and limitations

This study uses novel analytic methods to obtain a more complete understanding of disparities in the MI burden in Florida. Using SEBs age-adjusted MI mortality risks allows for adjustments for county-level sample size resulting in more stable estimates of MI mortality risks.

The use of a FSSS with a restricted likelihood ratio [20] results in the detection of both circular and non-circular clusters. Non-circular clusters would otherwise not be detected by the more common and widely used CSSS. Thus, use of FSSS reduces false negatives in cluster identification [64], and hence potentially results in better targeting of control efforts. Additionally, using a restricted log likelihood ratio test instead of log likelihood ratio limited the number of false positives, which also results in better targeting of preventive and control efforts.

This study is not without limitations. First, we chose to study counties rather than smaller geographic areas such as ZIP codes because the county is the smallest geographic area for which annual population estimates are available from the Florida Legislature’s Office of Economic and Demographic Research. The county is also more relevant to policy action steps. However, the choice of the county as the sampling unit means that study design is prone to ecologic fallacy. Thus, study findings need to be interpreted with caution, ensuring that all causal inferences are made at the county level and not at the individual level. Additionally, counties are heterogenous with respect to geographic, socio-demographic, and environmental factors, hence summarizing the data by county may have masked intra-county disparities in MI mortality risks, which could be large [65]. Therefore, local health planning could benefit from analyses at lower geographic units such as 5-digit zip code or Census tracts or blocks, and this study may be used to guide future small-area studies.

Secondly, there is potential for geographic variation in diagnosis and reporting of MI as the underlying cause of death, which could lead to misclassification bias [66]. Third, the study did not capture the full burden of MI mortality in Florida, since the analysis was limited to Florida residents as denominator data were not available to estimate the non-resident population.

Fourth, the study did not investigate the determinants of the observed spatiotemporal disparities in MI-mortality risks. Therefore, follow-up studies will need to identify those factors especially in the high-risk clusters, and to investigate the drivers of the worrisome trends reflecting a stagnation or even a decrease in rates of decline in MI mortality risks in parts of North Florida. Identification of these determinants would provide crucial information for planning and guiding future health policy and control programs for MI and other CVD with similar risk factors as MI. Moreover, investigations of counties within low-risk clusters may provide insights regarding the protective factors contributing to lower than expected MI mortality risks in those counties.

Fifth, due to rapidly changing demographic trends including population aging; changes in racial and ethnic composition of the population; shift in household and family structures; and rapid population growth, the study results may not accurately reflect the current reality in the State of Florida. Unfortunately, the most current MI mortality data were not available when the study was initiated.

Lastly, the use of the likelihood ratio test to identify low-risk clusters may have resulted in clusters with higher relative risks than would otherwise be obtained with the restricted likelihood ratio test. This implies that the disparities in MI mortality risks between high- and low-risk clusters could actually be larger than estimated. The methodology for detecting circular and non-circular spatial clusters within the FlexSCan software needs further development to mitigate this limitation.

## Conclusions

There was substantial progress in reducing the overall MI burden and disparities in MI mortality risks in Florida over time. However, there are persistent geographical disparities, with high-risk clusters occurring primarily in rural northern counties and low-risk clusters occurring exclusively in urban southern counties. Moreover, the reduction in MI death risks in the north lagged behind that in the south by at least 1.5 decades. Since counties within high-risk clusters account for a sizeable proportion of the total population in Florida, prevention and control strategies should be targeted to those counties to maximize efficiency and effectiveness of interventions geared towards reducing health disparities and improving health for all Floridians. Moreover, MI shares similar risk factors with other CVD such as stroke, hence these health conditions tend to have similar geographic distribution. Thus, public efforts targeting those counties we identified as having persistently high MI risks would address not only MI disparities but also stroke and several of their risk factors such as diabetes, high blood pressure, etc. Suffice it to say that it is critical that planning and public health programs need to be guided by empirical evidence such as findings from this study so as to better address issues of health inequity and improve health for all.

## Abbreviations

CHD: Coronary heart disease
CSSS: Kulldorff’s circular spatial scan statistics
CVD: Cardiovascular disease
DOH: Florida Department of Health
FSSS: Tango’s flexible spatial scan statistics
LRT: Likelihood ratio test
MI: Myocardial infarction
RD: Risk difference
RLRT: Restricted likelihood ratio test
RR: Relative risks
SEB: Spatial Empirical Bayes
SES: Socioeconomic status
